# Atypical scrapie in sheep from a UK research flock which is free from classical scrapie

**DOI:** 10.1186/1746-6148-5-8

**Published:** 2009-02-10

**Authors:** Hugh A Simmons, Marion M Simmons, Yvonne I Spencer, Melanie J Chaplin, Gill Povey, Andrew Davis, Angel Ortiz-Pelaez, Nora Hunter, Danny Matthews, Anthony E Wrathall

**Affiliations:** 1 Animal Services Unit, Veterinary Laboratories Agency, Woodham Lane, New Haw, Addlestone, Surrey, KT15 3NB, UK; 2 Pathology Department, Veterinary Laboratories Agency, Woodham Lane, New Haw, Addlestone, Surrey, KT15 3NB, UK; 3 Molecular Pathogenesis and Genetics Department, Veterinary Laboratories Agency, Woodham Lane, New Haw, Addlestone, Surrey, KT15 3NB, UK; 4 ADAS Arthur Rickwood, Mepal, Ely, Cambridgeshire, CB6 2BA, UK; 5 Centre for Epidemiology and Risk Analysis, Woodham Lane, New Haw, Addlestone, Surrey, KT15 3NB, UK; 6 The Roslin Institute, Neuropathogenesis Division, University of Edinburgh Royal (Dick) School of Veterinary Studies, Ogston Building, West Mains Road, Edinburgh, EH9 3JF, UK; 7 TSE Consultants affiliated to Veterinary Laboratories Agency, Woodham Lane, New Haw, Addlestone, Surrey, KT15 3NB, UK; 8 Pathology, Infectious Disease & Biosecurity, School of Veterinary Science, University of Queensland, St Lucia, QLD 4072, Australia

## Abstract

**Background:**

In the wake of the epidemic of bovine spongiform encephalopathy the British government established a flock of sheep from which scrapie-free animals are supplied to laboratories for research. Three breeds of sheep carrying a variety of different genotypes associated with scrapie susceptibility/resistance were imported in 1998 and 2001 from New Zealand, a country regarded as free from scrapie. They are kept in a purpose-built Sheep Unit under strict disease security and are monitored clinically and post mortem for evidence of scrapie. It is emphasised that atypical scrapie, as distinct from classical scrapie, has been recognised only relatively recently and differs from classical scrapie in its clinical, neuropathological and biochemical features. Most cases are detected in apparently healthy sheep by post mortem examination.

**Results:**

The occurrence of atypical scrapie in three sheep in (or derived from) the Sheep Unit is reported. Significant features of the affected sheep included their relatively high ages (6 y 1 mo, 7 y 9 mo, 9 y 7 mo respectively), their breed (all Cheviots) and their similar *PRNP *genotypes (AFRQ/AFRQ, AFRQ/ALRQ, and AFRQ/AFRQ, respectively). Two of the three sheep showed no clinical signs prior to death but all were confirmed as having atypical scrapie by immunohistochemistry and Western immunoblotting. Results of epidemiological investigations are presented and possible aetiologies of the cases are discussed.

**Conclusion:**

By process of exclusion, a likely explanation for the three cases of atypical scrapie is that they arose spontaneously and were not infected from an exterior source. If correct, this raises challenging issues for countries which are currently regarded as free from scrapie. It would mean that atypical scrapie is liable to occur in flocks worldwide, especially in older sheep of susceptible genotypes. To state confidently that both the classical and atypical forms of scrapie are absent from a population it is necessary for active surveillance to have taken place.

## Background

### TSE-free sheep required for research

The bovine spongiform encephalopathy (BSE) epidemic in the United Kingdom (UK) led to a need for the British government to commission research on transmissible spongiform encephalopathies (TSEs) in cattle, sheep and other species. It became clear, however, that so far as sheep were concerned, the possibility of scrapie being present in locally sourced sheep in the UK would compromise such research. Lack of definitive tests to detect scrapie infection in living animals, and the known long-term persistence of the infective agent in the environment [1] also meant that it would be difficult to guarantee any indigenous British sheep as being free from the disease. For this reason the Ministry of Agriculture, Fisheries and Food (MAFF), (which later became Department for Environment, Food and Rural Affairs (Defra)), decided to create a flock in the UK from which guaranteed scrapie-free sheep carrying a range of known scrapie-susceptibility genotypes could be supplied to other establishments for research on TSEs.

### Scrapie and genetic susceptibility

Classical scrapie is a TSE (prion disease) of sheep and goats which has been diagnosed in many countries of the world [2]. Atypical scrapie, a new form of TSE, was first described in Norway in 2003 [3]. Diagnosis of atypical scrapie is made if a) the Western blotting (WB) test on brain tissue reveals a recognisable protein band with a molecular mass of less than 15 kDa, and b) conspicuous PrP^sc ^immunolabelling is detected by immunohistochemistry (IHC) in the cerebellum and in the nucleus of the spinal tract of the trigeminal nerve in the medulla at the obex level, but is absent in the dorsal motor nucleus of the vagal nerve [4].

Three important polymorphisms of the *PRNP *gene associated with susceptibility of sheep to classical scrapie are located at codons 136, 154 and 171 [5]. With regards to atypical scrapie, genetic testing of Norwegian sheep affected with the strain referred to as Nor98, as well as unaffected sheep from the same flocks, revealed that those with genotypes most susceptible to classical scrapie seemed unaffected by this atypical strain [3]. Instead it transpired that atypical scrapie is often linked to the ARR and AHQ alleles which tend to be associated with moderate to marked resistance to classical scrapie [6]. In addition, sheep with atypical scrapie have often been found to carry another polymorphism, with phenylalanine (F), rather than leucine (L) at codon 141. Thus the haplotype AF_141_RQ tends to confer increased susceptibility to atypical scrapie [7-9].

### Importation and biosecurity of the flock

To achieve their objective MAFF/Defra contracted the Veterinary Laboratories Agency (VLA) to import approximately 1000 sheep from New Zealand. New Zealand was selected because of its widely recognised status as a country free from TSE diseases of animals, including scrapie [2]. In addition, New Zealand's cautious importation policy has minimised its own risk of importing animals or animal feed that might carry such diseases. Prior to the importation of sheep from New Zealand into the UK a purpose-built facility was constructed at an Agricultural Development and Advisory Service (ADAS; now known as 'ADAS UK Ltd.') arable farm in East Anglia. In this paper the facility is referred to as the 'Sheep Unit'. Selection of the location of the Sheep Unit was based on the knowledge that a) the sheep population in that area is among the lowest in the UK, b) no livestock had been kept on the land for at least the previous 34 years, and c) manure had not been applied to the fields for at least the same period.

An initial importation to establish the breeding flock took place in February 1998 and a second to expand the flock to approximately 800 ewes took place in July 2001. On both occasions a Boeing 747 was specifically chartered, enabling import of approximately 1000 sheep each time (1036 in 1998 and 967 in 2001). Precautions to maintain TSE-free status during their journeys included confinement of the sheep within their transit crates for the whole time from Auckland, New Zealand, to their delivery into the Sheep Unit. Some of the imported sheep were sent out from the Unit for research soon after importation clearance had been completed, but the remainder were used to establish the breeding flock.

Risk assessments were carried out before the Sheep Unit was established to identify potential sources of pathogens, including TSE agents, which might gain access to the flock and invalidate the TSE-free status of the sheep. Personnel working at or visiting the Unit, entry of vehicles, equipment, food, bedding, veterinary drugs, pests, wildlife and many other factors were taken into account. Standard operating protocols, were written, which cover all materials, equipment and personnel entering the Unit, and these have been consistently applied, as a biosecurity shield. The Unit operates to ISO 9001:2000 standard.

The sheep are also tested regularly for a range of specified diseases including maedi/visna, chlamydial enzootic abortion, border disease, ovine Johne's disease and caseous lymphadenitis. Food and bedding are obtained from pastures in the UK that are known to have been free from potential TSE contamination for at least 25 years, and certain dietary and pharmaceutical items, such as ewe milk replacer and vaccines, are brought in from New Zealand. All equipment is purchased new, dedicated to the Unit and remains in the Unit. All of the sheep are individually identified and detailed electronic records are kept of procedures and events within the Unit.

### Breeding within the Sheep Unit

The flock contains the three breeds: Suffolk, Cheviot and Poll Dorset. As there is no ARH allele in the flock, it is possible by breeding to produce only ten of the possible 15 PrP genotypes. To increase production of homozygous animals and to reduce unwanted genotypes, any heterozygous females are mainly used as recipients (surrogates) for homozygous embryos. Table 1 gives the numbers of ewes of different breeds and of different PrP genotypes (homozygous at codons 136, 154, 171) that were present in the Sheep Unit in May 2002, ten months after the second importation. From 2006 some of the breeding animals have had the full open reading frame sequenced identifying the haplotype AF_141_RQ in the Cheviot breed but not the Suffolk or Dorset breeds. Due to the numbers of sheep produced by the unit, and the cost, this technique it is not routinely performed.

**Table 1:** Numbers of ewes of different breeds by homozygous genotypes that were in the Sheep Unit in May 2002

**Genotype**	**Cheviot**	**Suffolk**	**Poll Dorset**
ARQ/ARQ	76	324	29

ARR/ARR	32	108	31

VRQ/VRQ	88	-	13

AHQ/AHQ	15	-	-

**Total**	**211**	**432**	**73**

Guaranteed scrapie-free sheep of the ten genotypes are routinely supplied for Defra-funded or Defra-approved research projects, thereby enabling the UK government's research objectives to be achieved. As of 1 January 2008, over 4,700 such sheep had been supplied for research purposes. The flock has also supplied control materials such as blood for research, and it has the potential to supply cryobanked semen and embryos as well.

The Sheep Unit is closed to all but sheep (and, if ever necessary, semen and embryos) sourced from New Zealand, so, where possible, a family breeding system is usually undertaken, with five unrelated ram lines being used in successive years (in each breed/homozygous genotype combination) to minimise inbreeding. This is facilitated by the collection, freezing and storage of semen within the Unit. In addition to natural breeding, artificial insemination and embryo transfer have taken place in all breeding seasons to enable researchers' demands for certain genotypes to be met.

### Health monitoring in the Sheep Unit

All casualties and most of the mature sheep culled from within the Sheep Unit are subjected to general post mortem examination at either VLA Weybridge or VLA Bury St. Edmunds, UK. The brains of young lambs are considered unlikely to provide evidence of classical scrapie infection if examined, but, apart from these, the brains of other casualties and flock management culls, and also those of many of the sheep sent out from the Unit for research, are subjected to statutory diagnostic tests for scrapie at VLA Weybridge, and material retained for future reference. In June 2006 a recently lambed homebred ewe (G320) at the Sheep Unit showed signs of blindness, general malaise and a tendency to walk in circles. She was unresponsive to treatment, and in September 2006 was culled after her lamb was weaned. Post mortem examination revealed that she had atypical scrapie. Subsequent investigations revealed two further cases of the same disease in sheep associated with the Unit which had been supplied to other establishments for research.

This report gives details of these three atypical scrapie cases, and of the investigations into the possible origins of the disease following their discovery.

## Results

At the time when the data were collected (2006) the total number of sheep recorded as having been resident in the Sheep Unit was 5676, of which 2003 (35.3 per cent) were imported from New Zealand and 3673 (64.7 per cent) were homebred within the flock. Of these, 808 adults (14.2 per cent) were still alive within the Unit; 164 were imported and 644 homebred. Others had been sent to other institutes for research (many still alive), and a small number (74) had gone to the abattoir for human consumption, or to the VLA as culls or casualties to be examined post mortem for scrapie and other diseases. A further breakdown is given in Table 2.

**Table 2:** Numbers of sheep at time of data collection in 2006 that were recorded as being at the Sheep Unit, or sent for research, or to an abattoir, or for examination at the Veterinary Laboratories Agency (VLA)

**Location/destination**	**Imported**	**Homebred**	**Total**
Alive at Sheep Unit	164	644	808

Sent for research	951	1545	2496

Sent to abattoir for slaughter	0	74	74

Culls/casualties sent to VLA	888	1410	2298

**Total**	**2003**	**3673**	**5676**

If the 808 sheep that were still alive in the Sheep Unit, and the 74 aged one year or less that had been sent (without being tested) to the abattoir, are deducted, the remaining 4540 represents the number of sheep potentially available for testing (see Table 3). However, test results (IHC, WB or both) were available for only 1041 (22.9 per cent). As many of the remaining animals had been used in TSE challenge studies at various institutes, samples were either unavailable for examination, in some cases because they were still alive, or were compromised by virtue of having succumbed to experimental challenge. With regards to age, only about 40 per cent of sheep with test results were four or more years old.

**Table 3:** Brain tissue samples tested from sheep of the ten different genotypes in or from the Sheep Unit

**PrP genotype**	**Potential no. of sheep of the genotype**	**Sheep with relevant test results (%)**
ARR/ARR	793	221 (27.87)

ARR/AHQ	6	1 (16.67)

ARR/ARQ	538	207 (38.48)

AHQ/AHQ	91	33 (36.26)

ARQ/AHQ	32	5 (15.62)

ARQ/ARQ	1651	302 (18.29)

ARR/VRQ	303	47 (15.51)

AHQ/VRQ	31	14 (45.16)

ARQ/VRQ	498	131 (32.11)

VRQ/VRQ	597	80 (13.40)

**Total**	**4540**	**1041 (22.93)**

Nevertheless, in addition to the index case G320, two further cases of atypical scrapie were detected and confirmed by use of IHC and WB. At the time of testing, these two additional cases were at other institutes, one having left the Sheep Unit six months after arrival from New Zealand, and the other left a few months before being culled, having been sent as a surrogate dam carrying a lamb for future research. Details of the three cases are summarised in Table 4. It should be noted that all three sheep were relatively old Cheviot ewes with ARQ/ARQ genotypes. Two of the three were homozygous F/F at codon 141 and the other was an F/L heterozygote. Only the index case, G320, showed clinical signs prior to death (see additional file 1).

**Table 4:** Details of atypical scrapie cases in sheep in (or from) the Sheep Unit.

	**Sheep G320**	**Sheep Y071**	**Sheep D337**
**Birth date (and place)**	5 May 2000 (Sheep Unit) Born to a surrogate ewe following embryo transfer.	1 September 1999 (New Zealand). Imported to UK July 2001	1 September 1997 (New Zealand). Imported to UK February 1998

**Breed**	Cheviot	Cheviot	Cheviot

**Sex**	Female	Female	Female

**Genotype**	AFRQ/AFRQ	AFRQ/ALRQ	AFRQ/AFRQ

**Breeding history**	Lambed February 2002; May 2003; April 2004; June 2006	Lambed May 2003, May 2005, April 2006; February 2007	Never bred

**Length of stay in Sheep Unit**	6 y 4 mo	5 y 2 mo – sent to Sardinia in January 2007	6 mo – sent Institute for Animal Health in September 1998

**Research project (if any) which this animal was on**	Not on a research project	Not on a research project, but sent to Sardinia to lamb	Blood transfusion in September 1999 from a sheep challenged with BSE

**Date of death**	5 Sept. 2006	19 June 2007	2 May 2007

**Age at death**	6 y 4 mo	7 y 9 mo	9 y 7 mo

**Clinical signs**	Malaise, circling & blindness, ataxia (see additional file 1)	None seen before death. Routine cull.	None seen before death. End of experiment cull.

**Immunocyto-chemistry**	Positive throughout the brain	Positive cerebellum, but medulla negative	Positive throughout the brain

**Western blot**	Cortex positive	Cerebellum positive	Brain weakly positive

**Figure 1:**
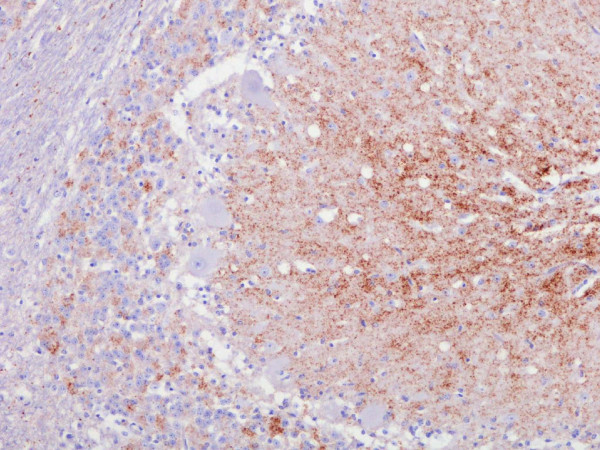
**Immunohistochemistry (IHC) of cerebellum from G320, showing widespread immunolabelling thoughout the molecular and granular layers of the cerebellum**.

Histopathological examination of the H&E stained sections of the obex (medulla at obex level) did not show vacuolar changes. However, in the brainstem of all three cases – the two examined by VLA Weybridge, and one (D337) which was part of an experimental group located at The Roslin Institute, Edinburgh and identified by VLA Lasswade (S. Siso, personal communication), IHC revealed accumulations of PrP^sc ^in the spinal tract nucleus of the trigeminal nerve, but not in the dorsal motor nucleus of the vagal nerve. PrP^sc ^was also demonstrated in the spinocerebellar tract in the case of G320. PrP^sc ^immunostaining in the cerebellum (Fig 1), was noted in all three cases, and was widespread throughout the brain for the two cases in which the material was available (G320 and D337).

**Figure 2:**
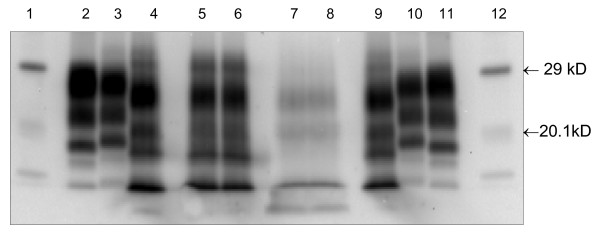
**Bio-Rad Western blot (WB) showing samples from G320 (frontal cortex) and Y71 (cerebellum) in duplicate lanes**. Controls are shown in single lanes as identified in the lane key below. The antibody used to detect PrP^sc ^was mAb Sha31 as provided in the test kit. Lane No.      Sample identity         Tissue type 1 and 12      molecular weight marker      marker 2 and 11      BSE +ve            control 3 and 10      classical scrapie +ve      control 4 and 9      atypical scrapie +ve   control 5 and 6      G320 (PG0832/06) (V1975B)   frontal cortex 7 and 8      YO71 (PG0604/07) (V2599C)   cerebellum

The molecular profile signals obtained using the 'TeSeE' (Bio-Rad) WB kit on samples from the three sheep were quite unlike those of classical scrapie or BSE, but they did resemble those of atypical scrapie (Nor98), with bands of molecular mass equal to or less than 15 kDa (Fig. 2). In the case of Y71 the signal was weaker than that of G320 whilst that of D337 was very faint and consequently has not been included in Fig. 2. The signal for D337 was, however, clearly visible using a different extraction procedure [10] at The Roslin Institute, and this will be reported fully elsewhere. Diagnosis of atypical scrapie in the three sheep was made based on the findings with IHC and the WB test.

For the two New Zealand-bred sheep which left the Sheep Unit, potential exposure to TSEs could not be ruled out. For Y71, however, such exposure could only have occurred during the 5 months between leaving the Unit in January 2007 and culling in June 2007 (Table 4). It is extremely unlikely that the pathology seen in Y71 could have developed in such a short interval. By contrast, D337 spent only 6 months at the Sheep Unit and the majority of its life on a project at the Institute for Animal Health (subsequently part of The Roslin Institute) where it was held in biosecure conditions. D337 was challenged with BSE as part of an experiment [11], however it survived the infection and was healthy at the time of death. If, in any of the three affected sheep reported here, another TSE had been present, it is likely that that TSE, rather than atypical scrapie, would have been evident from the IHC and WB test results.

## Discussion

It is noteworthy that a clinical presentation was evident only in the index case, G320, and the other two cases, Y71 and D337, were diagnosed retrospectively after they had been killed. The latter two had apparently been healthy immediately prior to their euthanasia and we do not know if they would have eventually developed clinical disease. The apparent blindness of sheep G320 prior to death was unusual in that previous cases of atypical scrapie have mostly presented with loss of body condition, sometimes with incoordination [12].

There are several possible origins of these atypical scrapie cases: exposure from the environment or infected animals (or, in the case of D337, from the experimental challenge), and spontaneous development of the disease within the sheep themselves. From our own investigations, and the currently available published evidence, none of these possibilities can be ruled out. We must point out, however, that if D337 had been infected by transfusion of blood from the BSE challenged sheep, we would have expected her IHC and WB test results to be characteristic of BSE rather than, as was the case, characteristic of atypical scrapie [4].

With regard to the possibility of exposure, we are aware that the most important risk factor for introduction of classical scrapie into a flock appears to be the purchase of infected animals [13] but, as yet, we do not know if this is also the case for introduction of atypical scrapie. Experimental transmission of atypical scrapie by intracranial inoculation of brain homogenate from affected cases has been demonstrated in transgenic (ovinised) mice [14], and in sheep with the AHQ/AHQ genotype which succumbed after a very variable incubation period ranging from 378 to 1057 days [[9]; unpublished observations]. This leaves open the question of whether the infection can be transmitted naturally between sheep or via a contaminated environment. To our knowledge, experimental transmission of atypical scrapie has not yet been achieved by the oral route, although studies are ongoing.

Our retrospective analyses and testing did not enable us to determine whether atypical scrapie infection was present among the sheep when they were imported from New Zealand or whether exposure and/or infection occurred after the Sheep Unit was established in the UK. With regard to the latter possibility, we believe that because the Sheep Unit is completely closed with strict biosecurity measures, the risk of introducing scrapie from the outside is very low indeed. At the time of writing (May 2008) only six cases of classical scrapie (and none of atypical scrapie) had been confirmed in the county (Cambridgeshire) in the past ten years, and all of these were beyond a 20 km radius of the Sheep Unit. Furthermore, all sheep and goats tested under the Fallen Stock and Abattoir surveys from holdings in Cambridgeshire have been negative. According to the 2005 agricultural census Cambridgeshire has less than 31 sheep per km^2^, which is one of the lowest densities in the UK, so contact between sheep in the Unit and other sheep is extremely unlikely. Nevertheless the Unit is not a high-security laboratory premises but a barriered isolation unit where humans (and occasionally wildlife such as birds and rodents) come into contact with its livestock. Therefore we cannot claim that sheep within the Unit are totally isolated from their environment. Comprehensive monitoring by IHC and/or WB is undertaken in animals culled from the flock, and also, where possible, through follow-up of animals leaving for other reasons. However, due to the age and genotype structure of the flock, only a relatively small number of sheep of genotypes that are now known to be at high risk of atypical scrapie [5,15] have been tested, and, of those that have been tested, few were aged four years and over.

Another possible explanation for the three cases of atypical scrapie is that they arose spontaneously and were not infected from an external source. The 'spontaneous aetiology' hypothesis for atypical scrapie in sheep has been mentioned by several authors [e.g. [7,10,16,17]] but there is no published evidence for it, and it would be difficult to test experimentally. In support of this hypothesis is the relatively consistent prevalence of atypical scrapie in national sheep flocks throughout the European Union (EU), despite significant variations in breed and management methods [15]. This suggests that atypical scrapie is potentially spontaneous at a consistent rate, or, alternatively, that it is poorly contagious.

Although atypical scrapie has been shown to be transmissible by experimental inoculation (see above), if it is a spontaneous genetic disease it may be similar in origin to the familial forms of TSE in man such as Gerstmann-Sträussler-Scheinker syndrome, Creutzfeldt-Jacob disease and fatal familial insomnia in which the resultant diseases can subsequently be transmitted experimentally [18,19]). In a recent article McIntyre [20] refers to the possibility of a spontaneous aetiology but also restates New Zealand's position as being free from classical scrapie and other TSEs of ruminants. For suppliers and users of TSE negative control sheep and cattle, and of TSE-free biological materials, the spontaneous aetiology hypothesis raises challenging nomenclature and certification issues. If the hypothesis is correct, one would expect sheep of susceptible genotypes in flocks across the world to be prone to develop atypical scrapie spontaneously, especially in old age. To state confidently that atypical scrapie is absent from a population, specific surveillance is required. It is not sufficient to have shown an absence of the classical disease because atypical scrapie has been shown to exist in sheep populations in which classical scrapie has not been detected [21,22]). Additionally, the low prevalence of atypical scrapie in the EU, and its widespread recognition only following the introduction of certain rapid tests into large surveillance programmes, argues that it may occur below the limit of detection even in countries that do carry out scrapie surveillance. As reported by Lühken et al. [17] and Moreno et al. [9], the AFRQ allele appears to confer the highest susceptibility to atypical scrapie, so the probability of detecting the disease is likely to be greatest in sheep of this genotype.

## Conclusion

In view of the fact that the three sheep affected with atypical scrapie reported here were carriers of the AFRQ allele, and were of relatively advanced ages, we are of the opinion that the spontaneous origin explanation is the one that should be given the most credence.

Disease monitoring, as outlined above, is continuing and the barriers to introduction of disease from outside the Sheep Unit will be maintained. However, with the low recorded incidence of atypical scrapie and the late age at onset, it will be a challenge to establish the true origin of the disease in this flock.

## Methods

Atypical scrapie was confirmed in the index case (G320) by the examination of samples of brain by histopathology, immunohistochemistry (IHC) and Western immunoblotting (WB). Subsequently a history of the flock was gathered from the database, including breeds, genotypes, parentage, source flock, siblings, offspring, and the various treatments such as vaccinations. Departure dates and destinations of sheep that had left the Unit were noted and, where possible, information about their fate was obtained, including date of death if this had occurred. Provided that experimental challenge had not compromised its suitability, samples of any archived brain tissues were also examined for scrapie. Particular emphasis was given to scrapie results from older sheep, i.e. those aged four years and over.

Brain tissues examined were primarily those from the obex but cerebral cortex and cerebellum tissue were used if obex was not available. These tissues were examined histopathologically and tested by IHC and by WB. PrP genotypes of all sheep were ascertained also.

For histopathology and IHC samples of fixed brain tissue were trimmed, embedded in paraffin wax and sectioned at 4 μm. Sections for histopathology were stained with haematoxylin and eosin (H&E). Detection of PrP^sc ^by IHC was performed using the mouse Mab 2G11 (Institute Pourquier) raised against the ovine PrP peptide sequence 146-R^154^R^171^-182, as previously described [23]. For WB samples of fresh (frozen-thawed) brain tissue were subjected to the 'TeSeE' sheep/goat Western blot (Bio-Rad Cat No: 355 1169) according to the kit instructions and using the reagents supplied. Essentially, tissue (0.35 g) from each sample was ribolysed, purified, proteinase K (PK) treated and PrP^sc ^concentrated. Samples were electrophoresed, transferred and the signal was detected with the 'Fluor-S MultiImager' (Bio-Rad). For genotyping of the suspect animals the full open reading frame of the ovine PrP gene was sequenced from samples of brain tissue, as previously described [8], in order to identify DNA polymorphisms and the resulting PrP protein amino acid changes at codons 136, 141, 154 and 171.

The sheep described in this paper were controlled under the UK Animal (Scientific Procedures) Act 1986 (project licence 70/6194, which was subject to routine ethical reviews).

## Abbreviations

ADAS: Agricultural Development and Advisory Service; BSE: bovine spongiform encephalopathy; CJD: Creutzfeldt-Jakob disease; Defra: Department for Environment, Food and Rural Affairs; EFSA: European Food Safety Authority; EU: European Union; H&E: haematoxylin and eosin; IHC: immunohistochemistry; LD: Local Detail; mAb: monoclonal antibody; MAFF: Ministry of Agriculture, Fisheries and Food; PK: proteinase K; PrP: prion protein; PrP^sc^: the scrapie- (disease-) associated isoform of PrP; TSE: transmissible spongiform encephalopathy; UK: United Kingdom; VLA: Veterinary Laboratories Agency; WB: Western immunoblotting.

## Authors' contributions

HAS is manager of the Sheep Unit project and had primary responsibility for the investigations reported here and for the manuscript; MMS had primary responsiblilty for interpretation of the histopathology and immunohistochemistry, especially of G320 and Y071; YIS was responsible for the immunohistochemical techniques and assisted with the interpretation of results of those; MJC was responsible for the Western blotting techniques and assisted with the interpretation of results of those; GP is responsible for flock management at the Sheep Unit and for data collection and retrieval; AD carried out pathology screening for the flock; AO-P carried out initial epidemiological investigations into the origin of the cases; NH is manager of the project in which the third case of the disease (D337) was found; DM co-ordinated all the data compilation and analysis following identification of the first case; AEW wrote the initial draft and, with DM, critically revised the manuscript. All authors contributed to and approved the final manuscript.

## Supplementary Material

Additional file 1 **Ewe G320 with lamb.** There is evidence of visual impairment (see ewe walking onto fence; this ewe also did not display a menace response) and general ataxia with loss of balance. Click here for file

## References

[B1] Georgsson G, Sigurdarsson S, Brown P (2006). Infectious agent of sheep scrapie may persist in the environment for at least 16 years. J Gen Virol.

[B2] Cosseddu GM, Agrimi U, Pinto J, Schudel AA (2007). Advances in scrapie research. Rev Sci Tech.

[B3] Benestad SL, Sarradin P, Thu B, Schönheit J, Tranulis MA, Bratberg B (2003). Cases of scrapie with unusual features in Norway and designation of a new type, Nor98. Vet Rec.

[B4] European Food Safety Agency (2005). Opinion of the scientific panel on biological hazards on the request from the European Commission on classification of atypical transmissible spongiform encephalopathy (TSE) cases in small ruminants. J EFSA.

[B5] Goldmann W (2008). PrP genetics in ruminant transmissible encephalopathies. Vet Res.

[B6] McIntyre KM, del Rio Vilas V, Gubbins S (2008). No temporal trends in the prevalence of atypical scrapie in British sheep, 2002–2006. BMC Vet Res.

[B7] Moum T, Olsaker I, Hopp P, Moldal T, Valheim M, Moum T, Benestad SL (2005). Polymorphisms at codons 141 and 154 in the ovine prion protein gene are associated with scrapie Nor 98 cases. J Gen Virol.

[B8] Saunders GC, Cawthraw S, Mountjoy SJ, Hope J, Windl O (2006). PrP genotypes of atypical scrapie cases in Great Britain. J Gen Virol.

[B9] Moreno CR, Moazami-Goudarzi K, Laurent P, Cazeau G, Andreoletti O, Chadi S, Elsen J-M, Calavas D (2007). Which PrP haplotypes in a French sheep population are the most susceptible to atypical scrapie?. Arch Virol.

[B10] Martin S, Gonzalez L, Chong A, Houston FE, Hunter N, Jeffrey M (2005). Immunohistochemical characteristics of disease-associated PrP are not altered by host genotype or route of inoculation following infection of sheep with bovine spongiform encephalopathy. J Gen Virol.

[B11] Houston F, McCutcheon S, Goldmnn W, Chong A, Foster J, Siso S, Gonzalez L, Jeffrey M, Hunter N Prion diseases are efficiently transmitted by blood transfusion in sheep. Blood.

[B12] Konold T, Davis A, Bone G, Bracegirdle J, Everitt S, Chaplin M, Saunders GC, Cawthraw S, Simmons MM (2007). Clinical findings in two cases of atypical scrapie in sheep: a case report. BMC Vet Res.

[B13] Green DM, del Rio Vilas VJ, Birch CPD, Johnson J, Kiss IZ, McCarthy ND, Kao RR (2007). Demographic risk factors for classical and atypical scrapie in Great Britain. J Gen Virol.

[B14] Benestad SL, Arsac JN, Goldmann W, Noremark M (2008). Atypical-Nor98 scrapie: properties of the agent, genetics and epidemiology. Vet Res.

[B15] Le Dur A, Beringue V, Andreoletti O, Reine F, Lai TL, Baron T, Bratberg B, Villotte JL, Sarradin P, Benestad SL, Laude H (2005). A newly identified type of scrapie agent can naturally infect sheep with resistant PrP genotypes. Proc Nat Acad Sci USA.

[B16] Hopp P, Omer MK, Heier BT (2006). A case-control study of scrapie Nor98 in Norwegian sheep flocks. J Gen Virol.

[B17] Lühken G, Buschmann A, Brandt H, Eiden M, Groschup MH, Erhardt G (2007). Epidemiological and genetical differences between classical and atypical scrapie cases. Vet Res.

[B18] Masters CL, Gajdusek DC, Gibbs CJ (1981). Creutzfeldt-Jacob disease virus isolations from the Gerstmann-Straussler syndrome with an analysis of the various forms of amyloid plaque deposition in the virus-induced spongiform encephalopathies. Brain.

[B19] Telling GC, Parchi P, DeArmond SJ, Cortelli P, Montagna P, Gabizon R, Mastianni J, Lugaresi E, Gambetti P, Prusiner SB (1996). Evidence for the conformation of the pathologic isoform of the prion protein enciphering and propagating prion diversity. Science.

[B20] McIntyre L (2007). New Zealand's contribution to explaining the pathogenesis of atypical scrapie. MAF Biosecurity New Zealand Surveillance Magazine.

[B21] Orge L, Galo A, Machado C, Lima C, Ochoa C, Silva J, Ramos M, Simas JP (2004). Identification of putative atypical scrapie in sheep in Portugal. J Gen Virol.

[B22] Epstein V, Pointing S, Halfacre S (2005). Atypical scrapie in the Falkland Islands. Vet Rec.

[B23] Simmons MM, Konold T, Simmons HA, Spencer YI, Lockey R, Spiropoulos J, Everitt S, Clifford D (2007). Experimental transmission of atypical scrapie in sheep. BMC Vet Res.

